# Late Quaternary activity of the Xiadian west concealed fault east to Beijing and its tectonic significance

**DOI:** 10.1371/journal.pone.0275436

**Published:** 2022-10-14

**Authors:** Jun Shen, Xunye Dai, Xuankai Jiao, Bo Shao

**Affiliations:** 1 China Institute of Disaster Prevention, Sanhe, China; 2 Hebei Key Laboratory of Earthquake Dynamics, Sanhe, China; 3 Institute of Science and Technology, China Tree Gorges Corporation, Beijing, China; Chinese Academy of Geological Sciences, CHINA

## Abstract

Beijing plain is a strong earthquake tectonic area in China. There was a Sanhe-Pinggu earthquake with Ms8 that happened in1679. The seismogenic fault of this earthquake is called Xiadian fault. Our work found fault with a similar strike and opposite dip in the west of the Xiadian fault, which is called the Xiadian west fault in this paper. Six shallow seismic profiles have been constructed to determine the location of the fault in Sanhe city, and the late Quaternary activity of the fault is studied with the method of combined drilling, magnetic susceptibility logging, and luminescence dating. The results of shallow seismic exploration show that the fault is zigzag and generally strikes NE and inclines NW. According to the core histogram and logging curves of ten boreholes and eight effective dating data, the buried depth of the upper breakpoint of the concealed fault is about 12 m, which dislocates the late Pleistocene strata. The effective dating result of this set of strata is 36.52 ±5.39 ka. The vertical slip rate has been about 0.075± 0.023 mm/a since the late Pleistocene and about 0.058 ± 0.030 mm/a since the late period of the late Pleistocene. It can be inferred that the Xiadian west fault is probably a part of the seismogenic structure of the Sanhe-Pinggu Ms8 earthquake that happened in 1679. In a broad sense, the Xiadian fault zone is likely to extend to the southwest along the Xiadian west fault.

## 1. Introduction

**Fig 1:**
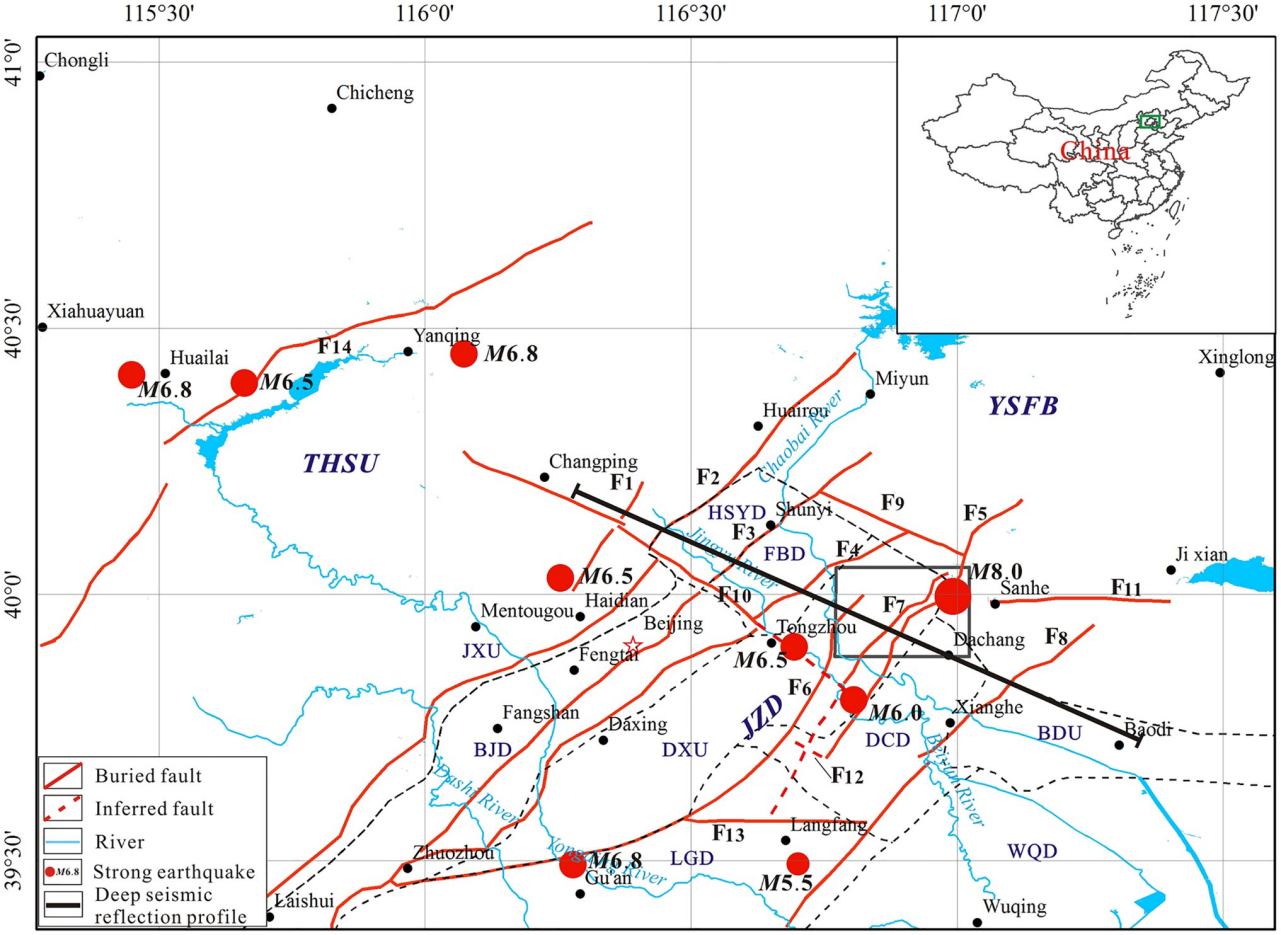
Location and buried faults distribution of the study area. The distribution of the faults is adapted from He et al. [28]. The tectonic units and their boundary are adapted from Gui et al. [33]. The seismicity data are from the China Earthquake Networks Center (CENC). F1 Xiaotangshan Fault; F2 Huangzhuang-Gaoliying Fault; F3 Shunyi-Liangxiang Fault; F4 Nanyuan-Tongxian Fault; F5 Xiadian Fault; F6 Daxing Fault; F7 Xiadian West Fault; F8 Xianghe Fault; F9 Ershilichangshan Fault; F10 Nankou-Sunhe Fault; F11 Pigezhuang Fault; F12 Yaoxinzhuang Fault; F13 Tongbai Fault; F14 Yanhuai Basin piedmont Fault. THSU: Taihangshan Uplift; YSFB: Yanshan Fold Belt; JZD: Jizhong Depression; JXU: Jingxi Uplift; BJD: Beijing Depression; DXU: Daxing Uplift; DCD: Dachang Depression; LGD: Langgu Depression; BDU: Baodi Uplift; WQD: Wuqing Depression; HSYD: Houshayu Depression; FBD: Fengbo Depression.

Several active faults have occurred in the Beijing plain. The Xiadian fault is one of the well-known active faults, which is the seismogenic tectonics of the Sanhe-Pinggu earthquake with Ms8.0 that happened in 1679 [1–6], Nankou Sunhe fault [7–9], Huangzhuang Gaoliying fault [10] (Fig 1). However, the more detailed structure of the Xiadian fault zone, such as whether there is a side branch fault, and the activity of the side branch fault in the late Quaternary are not clear.

**Fig 2:**
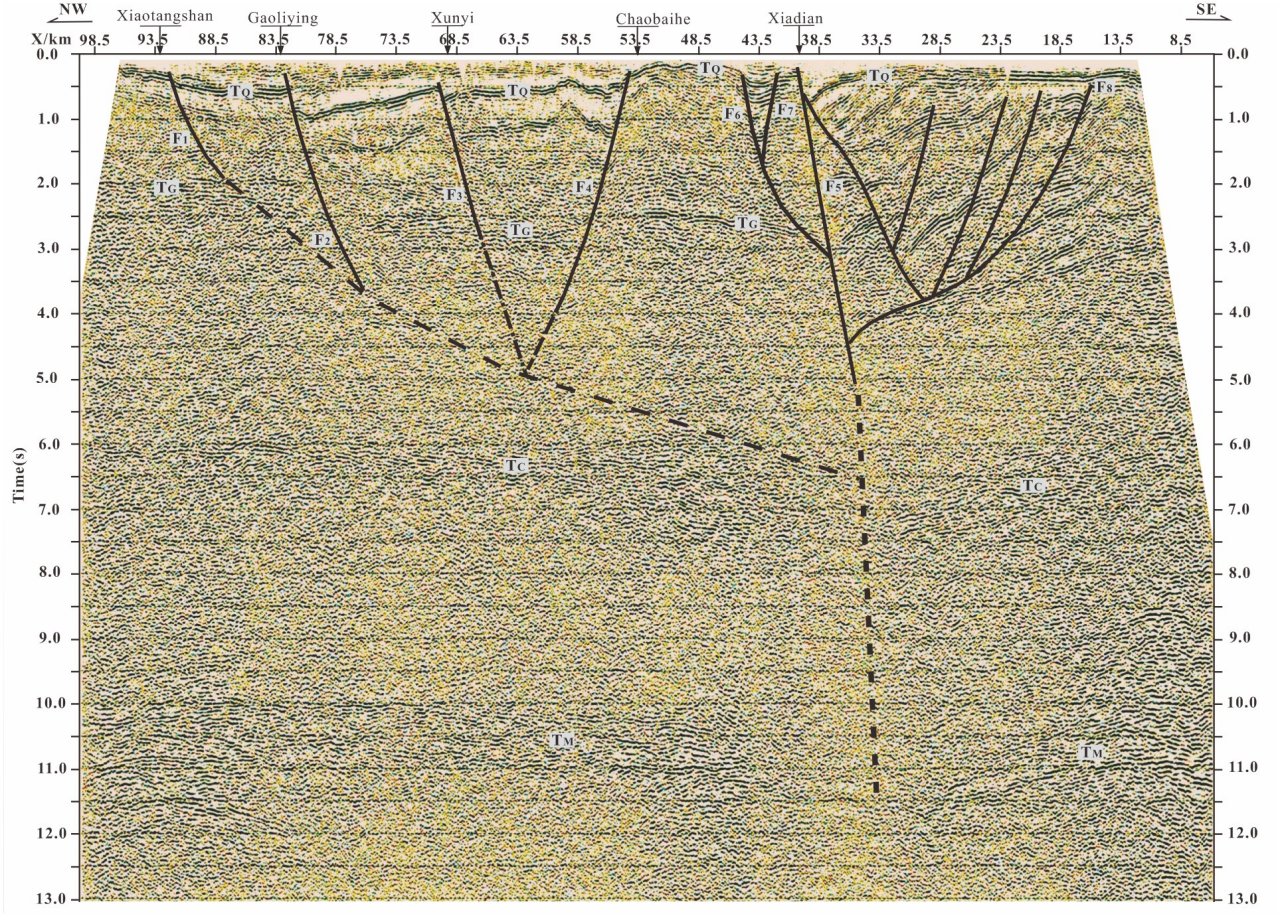
Deep fault structure in Beijing plain revealed by deep seismic reflection profile. The deep seismic reflection profile is adapted from Liu et al. [11], see Fig 1 for the Profile position. F1 Xiaotangshan Fault; F2 Huangzhuang-Gaoliying fault; F3 Shunyi-Liangxiang Fault; F4 Nanyuan-Tongxian Fault; F5 Xiadian Fault; F6 Daxing Fault; F7 Xiadian West Eault; F8 Xianghe fault; TQ bottom of Quaternary system; TG bottom boundary of sedimentary; TC Conrad discontinuity; TM: Moho discontinuity.

Xiadian fault is the seismogenic structure of the Sanhe Pinggu Ms8 earthquake in 1679. The deep seismic detection results show the direct exposure of the deep fault cutting through the whole crust on the surface (Fig 2) [11]. There is about 10 km seismic deformation zone on the surface. Many researchers have studied the Xiadian fault zone with different methods such as geology, micro geomorphology, geophysical prospecting, geochemical prospecting, and drilling. The macro epicenter, seismogenic mechanism, and fracture mode of the earthquake were studied [1,2,12–26]. A series of problems such as the nature, scale, and occurrence of the main fault in the Xiadian fault zone, regional tectonic stress field the characteristics, geological tectonic background, the repeatability of large earthquakes, and the activity characteristics since the late Pleistocene are discussed as well. However, Geological and geophysical data show that the length of Xiadian fault is only about 40km, while the seismogenic structure of a M8 earthquake is often greater than 100km [27]. Therefore, in addition to the Xiadian fault, it is likely that other faults together formed the seismogenic structure of this great earthquake. Therefore, it is necessary to find out whether there are active faults on the side of Xiadian fault. Up to now, it is rarely introduced whether there are active faults on the side of the Xiadian fault, and the southward extension of the Xiadian fault is not clear.

Previous studies have shown that the Daxing fault is developed to the west of the Xiadian fault [28–30] (Fig 1). Daxing fault, also known as Lixian-Niubaotun fault, eastern margin fault of Daxing uplift or northern margin fault of Gu’an basin, is the northwest boundary of the Langfang triangle’s large buried basin [28,29]. Petroleum Companies carried out intensive research on it, including seismic exploration and structural analysis, and obtained rich data and achievements about the fault’s geometry, kinematic characteristics, and information about sedimentary control of the Langfang sag [31–33].

According to Zhao et al. [34], in the north, it starts from the intersection of the NW trending fault in the east margin of Langfang sag and ends at the intersection of the NW trending Dongleiz-Laishui buried fault in the south with a total length of about 88 km. Zhang et al. [35] believe that the Daxing fault connects with the Xiadian fault, which controls the development of Jizhong depression. Xiadian fault is a Holocene Active fault on which the Pinggu-Sanhe earthquake of Ms8.0 happened in 1679 [2,3]. According to the historical records, there was a strong earthquake with Ms6.8 near the Daxing fault in 1057, and later in 1536; another strong earthquake with Ms6.0 occurred at the intersection of the Daxing fault and the Xiadian fault (Fig 1).

So far, the performance of the Daxing fault in the shallow strata of Neogene has not been studied. There are certain differences in the activity of the fault, which can be described from two viewpoints. The first one is that the fault is an early Middle Pleistocene fault, and the second is that the fault is connected with the Xiadian to the east, and there is an obvious local depression (300–500 m deep) along the fault zone. Comparing the relationship between the buried active faults and the thickness distribution of the Quaternary system, it is believed that this local depression had strong activity in the Quaternary, and the Daxing fault might be activated in late Quaternary.

He et al. [28] applied the shallow seismic method to study the activity of the northern segment of the Daxing fault, and concluded that the fault continued the extensional normal fault activity of Paleogene from Neogene to the early Quaternary period, and there was no activity since late Pleistocene. However, his explanation about the latest activity of the fault was not supported by the geological works. Based on high-precision shallow seismic exploration data and high-density composite drilling geological section surveys, Li et al. [29] consider the Daxing fault is a Holocene active fault. During our investigation on the active fault detection and seismic risk assessment in Sanhe City, we used shallow seismic exploration and row drilling detection methods to find the location and the late Quaternary activity of the fault between the Xiadian fault and Daxing fault, and uncovered a new approach. The process and results of this work are introduced below.

## 2. Geologic setting

The study area is located in the North China subsidence zone in the east of Beijing (Fig 1), and tectonically belongs to the Jizhong depression (JZD) of the North China Basin (NCB). It is connected to the Taihangshan uplift (THSU) in the west and the Yanshan fold belt (YSFB) in the north. Under the background of intense extensional rifting in the Paleogene Eocene to Oligocene of the Cenozoic era, the Beijing plain formed a tectonic pattern of alternating depressions and uplifts [2,28–29,36–38]. Macroscopically, they are distributed in the NNE direction and can be further divided into secondary structural units such as Jingxi uplift (JXU), Beijing depression (BJD), Daxing uplift (DXU), Dachang depression (DCD) Langgu depression (LGD), Wuqing depression (WQD), Baodi uplift (BDU) [29,39].

From the late Neogene to the Quaternary, the tectonic movement in the north china Basin was characterized by a continuous decline and acceptance of deposition, which made the Paleogene basin mountains and widely developed fault structures buried under thick Neogene and Quaternary sediments [2,40–42]. At the same time, due to the new NW-trending active faults, the structural features of the alternating depressions and uplifts formed since the Mesozoic in the Beijing Plain area began to disband. The direction of the long axis of the depositional center changed from the NNE direction of the Paleogene to the NW direction, forming new depositional centers such as Fengbo depression(FBD) and Houshayu depression(HSYD) [29,39].

The seismogenic faults in the Beijing Plain can be classified into two groups based on the general trend of the fault zone. These faults were formed in the Yanshanian period, and most of them have inherited activities since the Cenozoic. The north NE active fault has the most enormous scale, strong zonation and extensive activity range, and is closely related to seismic activity. It is the main active fault in the Beijing area. The NW strike faults have been active since Quaternary, and most of them are new faults, and their mechanical properties are primarily tensional and torsional [43]. The NW strike structure is mainly composed of the Nankou-Sunhe fault and the Ershilichangshan fault. The NNE-NE strike fault includes Xiaotangshan fault, Huangzhuang -Gaoliying fault, Shunyi-Liangxiang fault, Nanyuan-Tongxian fault, Daxing fault, Xiadian west fault, Xiadian fault and Xianghe fault.

## 3. Methods

Implementation of shallow seismic exploration combined with row drilling detection is the most effective method used to determine the fault location and the buried depth of the upper breakpoint and identify the activity of the late Quaternary concealed faults. In this paper, the activity of the Xiadian west fault in Sanhe City has been studied using the above method. The process is generally described as follows:

### 3.1 Shallow seismic exploration

**Fig 3:**
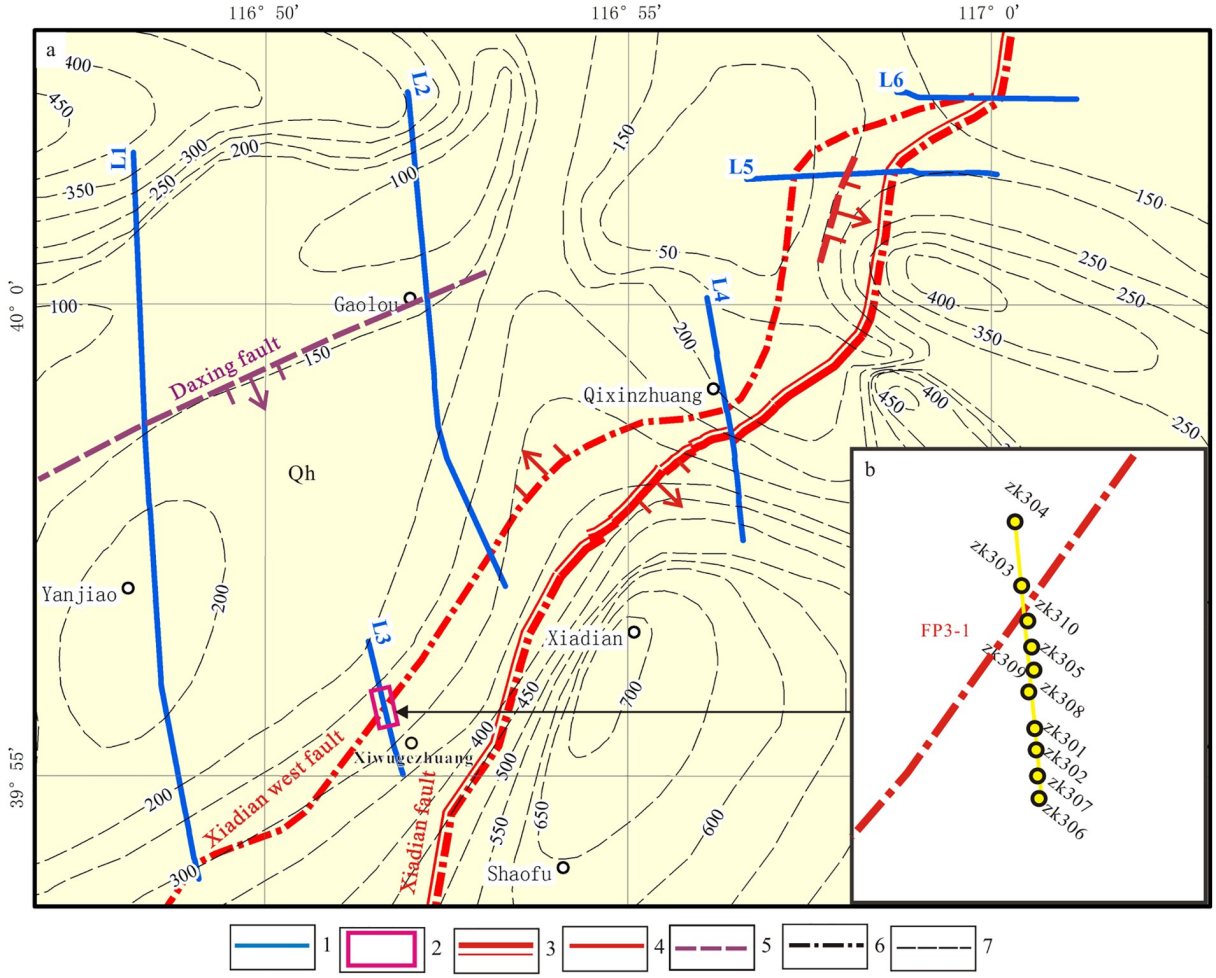
Location of shallow seismic exploration line and joint drilling profile detection(a) and borehole distribution of joint drilling profile detection (b). The Quaternary thickness contour is adapted from Deng et al. [4]. 1. Location and number of shallow seismic exploration lines; 2. Location of joint drilling profile; 3. Holocene fault; 4. Late Pleistocene fault; 5. Buried fault; 6. Surface fault; 7. Quaternary thickness contour.

As mentioned above, shallow seismic exploration is an effective technology to detect concealed active faults. Six shallow seismic exploration lines are laid to find out the location and activity of the Xiadian west fault, which is a newly discovered active fault between the Xiadian fault and the Daxing fault. The location of the lines is shown in Fig 3a, and the coordinations of the lines are shown in Table 1. Among those, L1 and L2 are long lines to investigate if there are any unknown active faults in the northwest of the Xiadian fault, which is considered the seismogenic tectonics of the Sanhe-Ping Ms8 earthquake that happened in 1679. The purpose of L3, L4, L5, and L6 is to find out the exact location of the potential active fault and its relationship with the Xiadian fault.

**Table 1:** Coordinates of shallow seismic exploration lines.

Line number	Starting point coordinates			End point coordinates		
	Point number	longitude	latitude	Point number	longitude	latitude
L1	100	116°48′10.37″	40°1′36.77″	14558	116°49′5.82″	39°53′54.21″
L2	100	116°51′57.21″	40°2′15.47″	10190	116°53′18.71″	39°57′0.88″
L3	100	116°50′15.53″	39°55′47.56″	3000	116°50′35.89″	39°54′15.67″
L4	2234	116°56′5.16″	40°0′4.69″	7080	116°56′35.05″	39°57′30.20″
L5	100	116°56′37.87″	40°1′20.09″	5078	117°0′4.97″	40°1′23.06″
L6	100	116°58′42.32″	40°2′15.44″	3674	117°1′10.67″	40°2′11.11″

#### 3.1.1 Data acquisition

In this shallow seismic exploration technique, M18–612 Vibroseis made in the United States and Sercel-SN388 Digital Seismograph made in France are used for data acquisition by maintaining specific setuo, i.e., 1 ms sampling interval, 2.0s recording length, and 60Hz high-frequency geophone reception. The observation system is composed of track spacing of 2 m, shot spacing of 12 m, offset of 12 m, 240 track arrangements, unilateral excitation, and 20 coverage times.

#### 3.1.2 Data processing

The collected data are processed by ① preprocessing, ② refraction static correction, ③ surface consistent deconvolution, ④ FK filtering, ⑤ fine velocity analysis, ⑥ residual static correction, ⑦ noise attenuation, and ⑧stacking and migration to obtain the stacking profiles with high signal-to-noise ratio and clear wave group characteristics.

#### 3.1.3 Profile interpretation

*1) Identification of reflection horizon*. During the active fault detection in Sanhe city, the analysis of regional geological data, followed by a combination with the data of standard reference hole is carried out. The seismic reflection profile in the study area is divided into eight groups of reflection waves according to their specific characteristics. The bottom boundary of the Quaternary system is taken as the mark layer, and the number is T_Q_. The horizon above T_Q_ is marked as T_01_, T_02_ and T_03_ from top to bottom. The strata below T_Q_ are marked as T_11_, T_12_, T_13_ and T_14_ from top to bottom. There is an unconformity interface between the Cenozoic and the previous old strata, and the seismic reflection energy is strong. The seismic lines in the study area can be identified and marked as T_14_. Compared with the data of standard holes carried out during the active fault detection in Sanhe City, Q_p3_ corresponds to T_01_, Q_P2_ corresponds to T_02_, and Q_p1_ corresponds to T_Q_. T_03_ is the Q_p1_ internal reflection wave.

*2) Fault identification*. During stack section, the fault is inferred and interpreted according to the reflection wave’s characteristics of the seismic reflection section and the geological structural characteristics of the study area. The main basis of the fault discrimination in data interpretation is as follows:

① The obvious interruption and dislocation of the reflection phase axis.
② The increase or decrease in the number of in-phase axes of the reflection wave.
③ The sudden change in the energy and in-phase axis shape of the reflected wave.
④ The strong phase inversion of the reflection wave’s in-phase axis and the mutual dependence of upper and lower wave groups.
⑤ The wave group changes and there are obvious dislocations.
⑥ Although the in-phase axis can be traced continuously, the wave group changes as a whole.
⑦ The appearance of an abnormal wave is detected (such as diffraction wave, cross-section reflection wave, etc.).
⑧ There are obvious differences in the occurrence between the upper and lower walls of the fault.

*3) Time depth conversion*. The average velocity above different strata interfaces is calculated according to the stack velocity obtained during the data processing and the two-way vertical travel time t_0_ of the reflection wave from different interfaces on the time profile.

### 3.2 Row drilling profile detection

In order to find out the activity of the fault in the late Quaternary, a row of drill holes was set up at the location of Fp3–1 at point No. 750 on shallow seismic exploration line L3. The profile is located in Xiwugezhuang, Sanhe City. The borehole coordinations and depth are shown in Table 2, and the borehole layout is shown in Fig 2b.

**Table 2:** Coordinations and depths of the combined row drilling holes at Xiwugezhuang on L3 shallow seismic exploration line.

Borehole number	Coordinates(WGS84)		Borehole depth(m)
	latitude	longitude	
Zk304	39°54’38.6"N	116°50’33.5"E	83.80
Zk303	39°54’37.6"N	116°50’33.6"E	77.20
Zk305	39°54’36.4"N	116°50’33.9"E	67.30
Zk308	39°54’36.1"N	116°50’33.8"E	45.60
Zk301	39°54’35.8"N	116°50’33.9"E	66.50
Zk302	39°54’35.2"N	116°50’34.0"E	73.80
Zk307	39°54’34.8"N	116°50’34.0"E	50.50
Zk306	39°54’34.5"N	116°50’34.0"E	50.80
Zk309	39°54’36.4"N	116°50’33.8"E	27.60
Zk310	39°54’37.2"N	116°50’33.7"E	36.80

#### 3.2.1 Drilling layout and construction

*1) Drilling layout*. The basic idea is to use the folding method to gradually lay out boreholes across the fault and approach the middle. In the specific construction, due to the presence of many suspected faults, the upper breakpoint of the fault becomes shallower than the fault inferred by shallow seismic exploration; the occurrence of the fault becomes slower along with many breakpoints. In the actual construction, the scheme has been adjusted in two ways, one is to expand the section to the south, and the other is to infill drilling.

*2) Drilling construction*. XY-200 drilling rig is used in this drilling work, with hole diameter of 146 mm and coring diameter of 110 mm; clay slurry is used to protect the wall. Core recovery: 90% for clay and silt cores, 80% for medium-fine sand, and no less than 40% for loose coarse sand. The hole depth, footage, core length and recovery rate of the core are indicated, and the core is put into the core preservation tube from top to bottom in order.

#### 3.2.2 Core logging

Firstly, the rock core column is photographed completely. The basic logging unit is determined according to lithology, color, material composition, sedimentary structure, and contact interface shape. Next, the elemental map and the text description are created. The contents of logging include, ① stratigraphic sequence, thickness and depth; ② stratigraphic color; ③ grain size and percentage of the components of different grain sizes; ④ clastic composition, morphology and roundness; ⑤ stratigraphic cementation degree and bedding structure characteristics; ⑥ mineral nodules, animal fossils and plant fossils; ⑦ layered contact relationship; ⑧special phenomena such as structural deformation and rapid abnormal accumulation; and ⑨ age sample’s acquisition location.

The field work of core logging starts from pulling out the core by the drilling rig until the sketch of the drilling histogram is drawn. The specific logging process is as follows: a) determining the top and bottom of the core; b) cleaning the mud on the surface of the core using special tools; c) measuring the length of the core [the actual depth of a layer of core = the depth of the previous layer + (footage/core length) × the thickness of the layer of the core]; E) description of the core; F) take photos of the core; g) drawing a sketch of the drilling column.

#### 3.2.3 Logging

If the logging process of the core histogram is performed only by visual observation and qualitative description ineluctable errors and uncertainties appear in the judgment results of faults and dislocations. In order to reduce the artificial error and uncertainties, the logging work has been carried out based on the above manual logging process.

*1) Logging equipment*. Geovista integrated logging instrument from Beijing Judeng Geophysical Exploration Company is used in this logging work to measure the apparent resistivity, natural gamma, and magnetic susceptibility. Geovista’s susceptibility probe (commonly known as MAGS) is mainly used to explore magnetite, pyrite and hematite deposits. It is used to detect and determine the dislocation amount of concealed faults.

*2) Logging process*. After completing each borehole, clean water is used to change the slurry to ensure the accuracy and reliability of the logging data. After connecting the instrument and putting on the power button, the controller adjusts the winch to lower the probe; at the same time, the Geovista release6.66 acquisition system is opened to start the measurement. The data processing software of the equipment is used to process the data and form the magnetic susceptibility curve for each borehole.

#### 3.2.4 Dating sample collection and testing

According to the requirements of dating sample collection for the optically stimulated luminescence (OSL), dating method samples were collected from ZK301 and ZK303 of the row drilling. The samples are tested in the Laboratory of Neotectonic Geochronology of the Institute of Disaster Prevention Science and Technology, CEA, by using the Fine Quartz(FQ), Simplified Multiple Aliquot Regenerative-dose protocol(SMAR) method. The results are shown in Table 3.

**Table 3:** The test results of dating samples.

Sample number	Depth (m)	U-238 (Bg/Kg)	Th-232 (Bg/Kg)	K-40 (Bg/Kg)	Water content(%)	dose rate (Gy/ka)	Equivalent dose(Gy)	Age(ka)
ZK301-osl1	3.50	17.42 ±1.56	41.16 ±4.39	662.89 ±0.73	19	2.91 ±0.1	44.47 ±4.11	15.3 ±2.11
ZK301-osl5	32.80	36.86 ±29.6	38.17 ±4.66	784.53 ±16.48	21	3.01 ±0.1	273.17 ±31.99	90.64 ±14.10
ZK301-osl6	42.50	75.5 ±5.21	47.54 ±4.37	598.8 ±10.24	15	3.03 ±0.1	401.34 ±22.18	132.64 ±15.44
ZK303-osl1	3.15	49.41 ±0.346	48.48 ±2.37	746.14 ±10.22	7	3.57 ±0.1	41.36 ±2.89	11.58 ±1.44
ZK303-osl3	15.35	29.47 ±3.28	37.81 ±2.92	677.15 ±18.28	4	3.28 ±0.1	119.88 ±12.71	36.52 ±5.39
ZK303-osl6	30.85	68.06 ±8.66	46.34 ±3.34	800.45 ±18.48	12	4.21 ±0.1	231.68 ±16.33	59.74 ±7.43
ZK303-osl10	50.15	26.17 ±13.14	39.33 ±3.88	758.05 ±7.28	4	3.54 ±0.1	396.59 ±53.91	112.00 ±19.07
ZK303-osl12	55.95	72.12 ±4.48	45.05 ±2.39	759.66 ±8.43	16	3.85 ±0.1	454.65 ±51.76	118.20 ±18.10

## 4. Results and analysis

### 4.1 Shallow seismic exploration results and analysis

**Fig 4:**
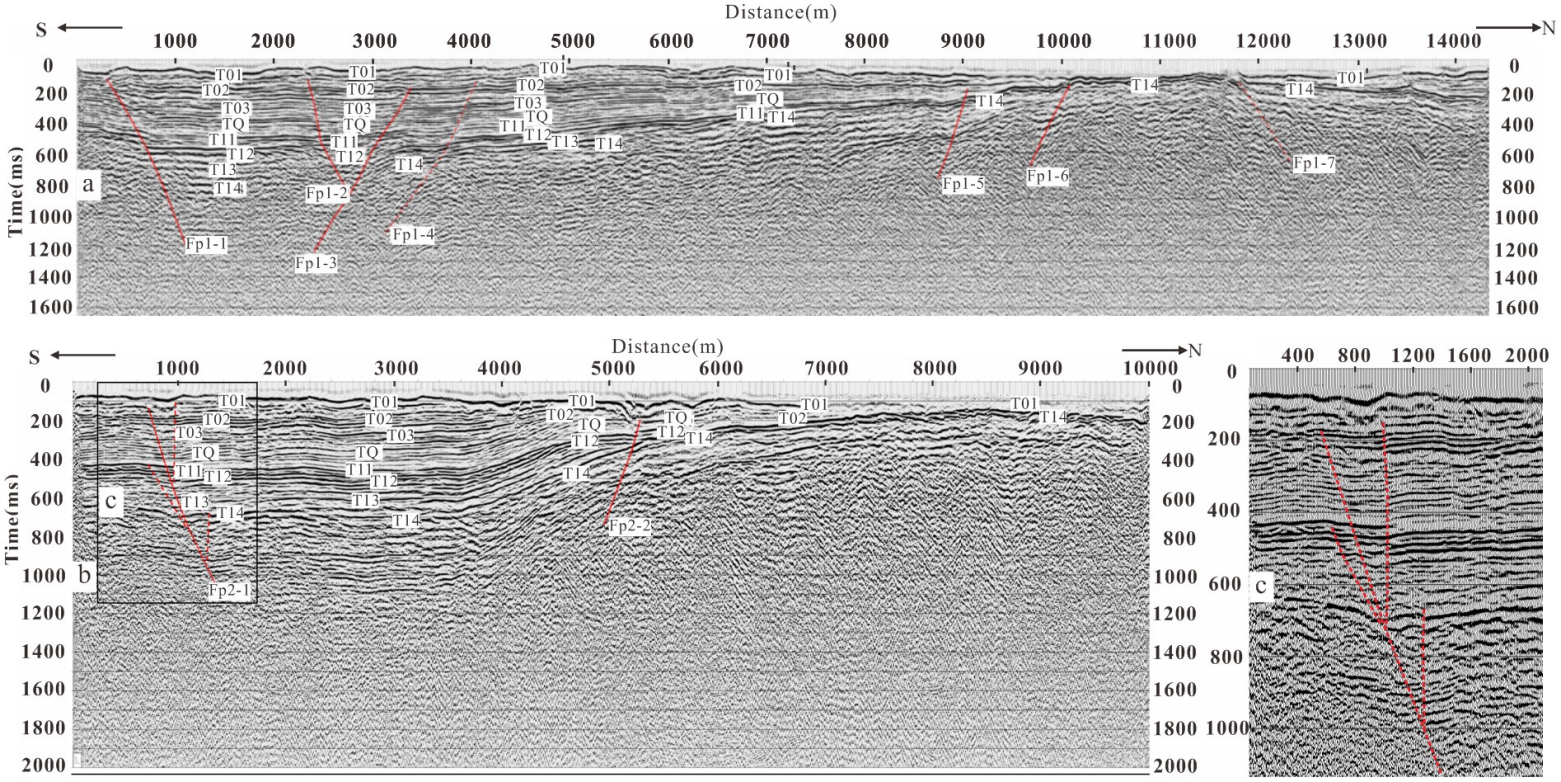
Seismic reflection time profile and geological interpretation of L1 and L2 exploration lines. (a) The seismic reflection migration time profile of the L1. (b) The time profile of the seismic reflection migration of the L2. (c) A local enlargement of the fault Fp2-1 in Fig 4b.

**Fig 5:**
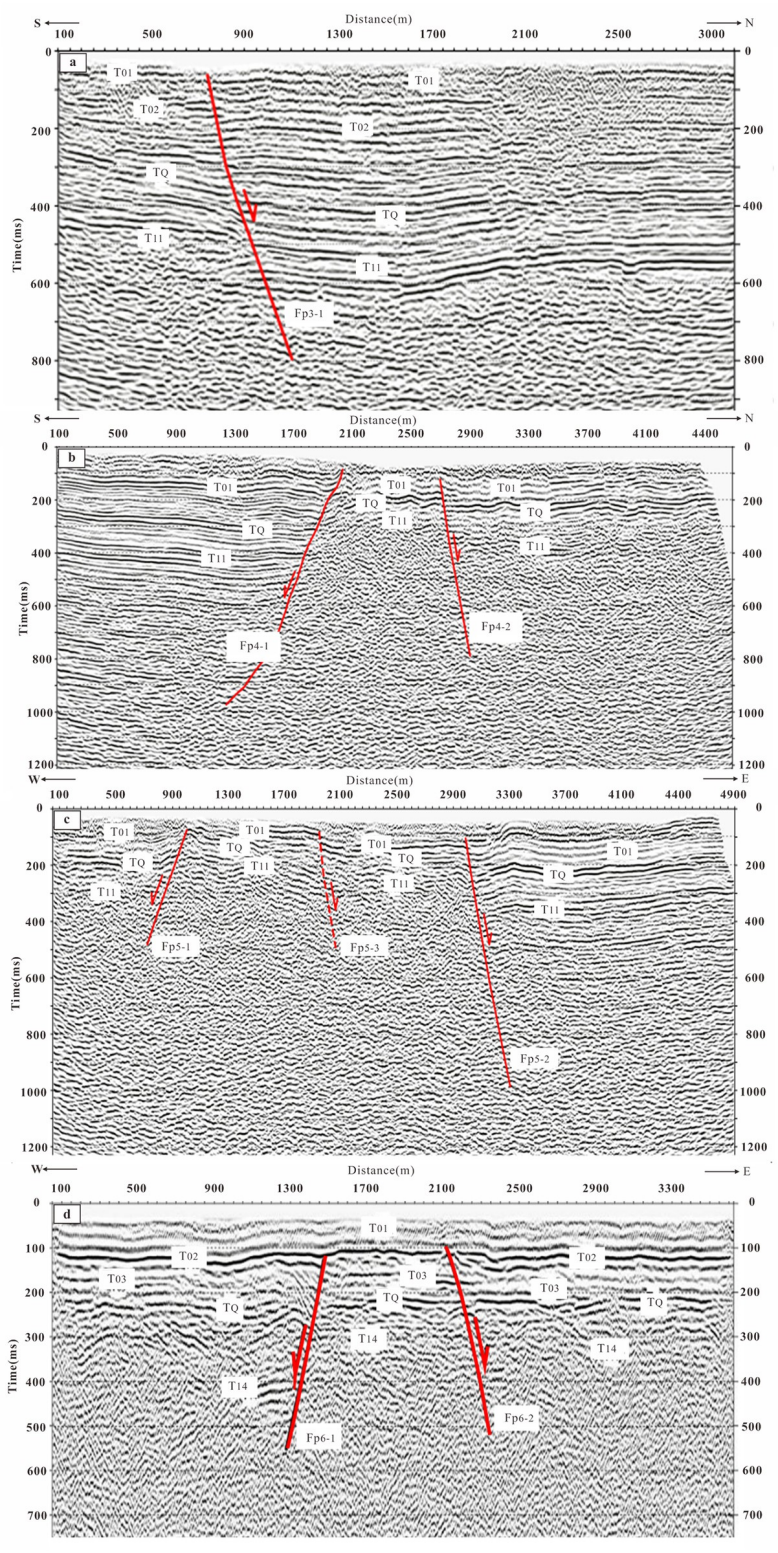
Seismic reflection time profile and geological interpretation of L3, L4, L5 and L6 exploration lines. (a) The time profile of seismic reflection migration of the exploration L3. (b) The time profile of seismic reflection migration of the exploration L4. (c) The time profile of seismic reflection migration of the exploration L5. (d) The time profile of seismic reflection migration of the exploration L6.

The reflection time profiles and geological interpretation profiles of the shallow seismic exploration lines are shown in Figs 4 and 5. The analysis and interpretation of each profile are as follows:

#### 1) L1 line

Fig 4a shows the seismic reflection migration time profile of the line, and the bottom figure of Fig 3 shows the geological profile obtained by time-depth conversion. There are eight groups of reflected waves with obvious characteristics labeled as T_01_, T_02_, T_03_, T_Q_, T_11_, T_12_, T_13_ and T_14_.

Eight faults are interpreted on the profile according to the characteristics of the reflection wave group and the basis of the fault difference. F_p1–1_, F_p1–2_ and F_p1–7_ incline to the north, and the other five faults incline to the south. The projection of the upper breakpoint of F_p1–1_ on the ground is located at CDP 300 m of the exploration line, with a buried depth of about 77 m. F_p1–1_ dislocates T_02_ and T_14_ vertically by about 13 m and 65 m, respectively. The buried depth of the upper breakpoint of F_p1–2_ is about 80 m. F_p1–2_ vertically dislocates T_02_ and T_14_ about13 m and 26 m, respectively. The buried depth of the upper breakpoint of F_p1–3_ is about 135 m. F_p1–3_ vertically dislocates T_02_ and T_14_ by about 12 m and 35 m, respectively. The buried depth of F_p1–4_ is about 116 m. It offsets T_02_ by about 4 m. The buried depth of the upper breakpoint of F_p1–5_ is about 140 m, and the T_Q_ is vertically offset by about 10 m by it. The buried depth of the upper breakpoint of F_p1–6_ is about 110 m. T_14_ is vertically offset by about 14 m by F_p1–6_. The buried depth of the upper breakpoint of F_p1–7_ is about 93 m, while T_14_ is vertically offset by about 8 m by F_p1–7_.

FP1–1 is the main fault among all the eight faults, which is judged to be a newly found fault between the Xiadian fault and Daxing fault. We named it the Xiadian west fault in this paper. Its northern part contains a dustpan-like depression or half-graben with deep in the south and shallow at the north. F_P1–5_ and F_P1–6_ are a northern segment of the Daxing Fault.

#### 2) L2 line

Fig 4b shows the time profile of the seismic reflection migration of the line. The time profile of the seismic migration is less than 700 ms, and 8 groups of reflection waves with obvious characteristics are interpreted, which are marked as T_01_, T_02_, T_03_, T_Q_, T_11_, T_12_, T_13_ and T_14_, respectively.

Two faults, F_p2–1_ and F_p2–2_, are interpreted on the profile according to the characteristics of the reflection wave group and the basis of fault discrimination. F_p2–1_ inclines to the north, the projection of the upper breakpoint on the ground is located near CDP706 m on the exploration line. The time is 138 ms, and the buried depth is about 94 m. The T_02_ and T_14_ are vertically offset by the fault at about 7 m and 20 m, respectively. F_p2–2_ inclines to the south, the buried depth of its upper breakpoint is about 105 m, and the T_02_ is vertically offset about 10 m by it. The two faults control a graben basin and the overall characteristics are similar to that L1 survey line. F_p2–1_ is the Xaidian west fault, and F_p2–2_ is the Daxing fault. Fig 4c shows the Local features of F_p2–1_, which has a typical flower like structure indicating the characteristics of strike slip fault.

#### 3) L3 line

The survey line is located in the east of line L1 to find out the finer structure of the Xiadian west fault(Fig 3a). Fig 5a shows the time profile of seismic reflection migration of the exploration line. Four groups of reflection waves with significant characteristics are interpreted with the depth of two-way travel-time of about 600 ms, which are marked as T_01_, T_02_, T_Q_ and T_11_, respectively. The fault Fp3–1 is interpreted, which inclines to the north and is a normal fault. The vertical projection of the upper breakpoint on the ground is about GDP750 m on the exploration line, the time is about 60 ms, and the buried depth is about 30 m. The vertical offsets of the T_02_ and T_Q_ are about 12 m and 66 m, respectively.

#### 4) L4 line

This exploration line crosses the Xiadian fault and Xiadian west fault. Fig 5b shows the seismic reflection migration time profile of the line. Eight groups of reflected waves with specific characteristics are interpreted, which are labeled as T_01_, T_02_, T_03_, T_Q_, T_11_, T_12_, T_13_ and T_14_. Two faults are interpreted on the profile. F_p4–1_ fault inclines to the south, the vertical projection of the upper breakpoint on the ground is located near the GDP4068 m, the buried depth is about 50 m with the two-way travel-time about 70 ms. At this depth, the T_02_ is vertically offset by about 10 m. The T_Q_ is vertically offset by about 45 m. The F_p4–2_ fault shows a northward dip on the profile. The vertical projection of the upper breakpoint of the fault on the ground is about GDP4844 m of the exploration line with a two-way travel time of about 120 ms and a buried depth of about 86 m. At this depth, T_02_ is vertically offset by about 9 m and T_Q_ is vertically offset by about 27 m. Fp4–1 is the Xiadian fault, and Fp4–2 is the Xiadian west fault. The distance between them is only about 600 m.

#### 5) L5 line

This line also crosses the Xiadian fault and Xiadian west fault. Fig 5c shows the time profile of seismic reflection migration of the line. The reflection waves with specific obvious characteristics are explained in 800 ms, which are marked as T_01_, T_02_, T_03_, T_Q_, T_11_, T_12_, T_13_, and T_14_. Three faults are explained on the profile. Among them, Fp5–1 inclines to the west. The vertical projection of the upper breakpoint on the ground is located near the GDP1038 m of the exploration line, with a two-way travel-time of about 81 ms and a buried depth of 48 m. The T_02_ is vertically offset by about 4 m, and T_Q_ is vertically offset by about 30 m. F_p5–2_ inclines to the east. The vertical projection of the upper breakpoint of Fp5–2 on the ground resides near the GDP3034 m of the exploration line, with a two-way travel time of about 104 ms and a buried depth of 60 m. The T_02_ and T_Q_ are vertically offset by about 9 m and 35 m, respectively. Fp5–3 also inclines to the east. The vertical projection of the upper breakpoint of it on the ground is located near the GDP1922 on the exploration line, with a two-way travel time of about 80 ms and a buried depth of 50 m. The T_02_ and T_Q_ are vertically offset by the fault about 4 m and 13 m, respectively. According to the fault profile and the breakpoint position, Fp5–1 has been considered the Xiadian west fault. Fp5–2 is the main fault of the Xiadian faut, and Fp5–3 is a branch fault of the Xiadian fault.

#### 6) Line L6

Fig 5d shows the time profile of seismic reflection migration of the line. The seismic migration time profile is less than 400 ms. Five groups of reflection waves with specific characteristics are interpreted, which are marked as T_01_, T_02_, T_03_, T_Q_, and T_14_, respectively. Two faults are explained on the profile. Fp6–1 inclines to the west, and the vertical projection of the upper breakpoint on the ground is near the GDP1518 m with a two-way travel time about 130 ms, and the buried depth is about 95 m. The vertical offset of T_02_ and T_Q_ are about 20 m, and 25 m, respectively. Fp6–2 inclines to the East, and the vertical projection of the upper breakpoint on the ground is near the GDP 2156 m with a two-way travel time of about 97 ms and a buried depth of 71 m. The vertical offsets of T_02_ and T_Q_ are about 7 m and 15 m, respectively. Through the analysis of fault profile figures, it is considered that fp6–1 is the Xiadian west fault, and Fp6–2 is the Xiadian fault.

### 4.2 Exploration results and analysis of Row drilling profile

#### 4.2.1 Stratigraphic analysis

**Fig 6:**
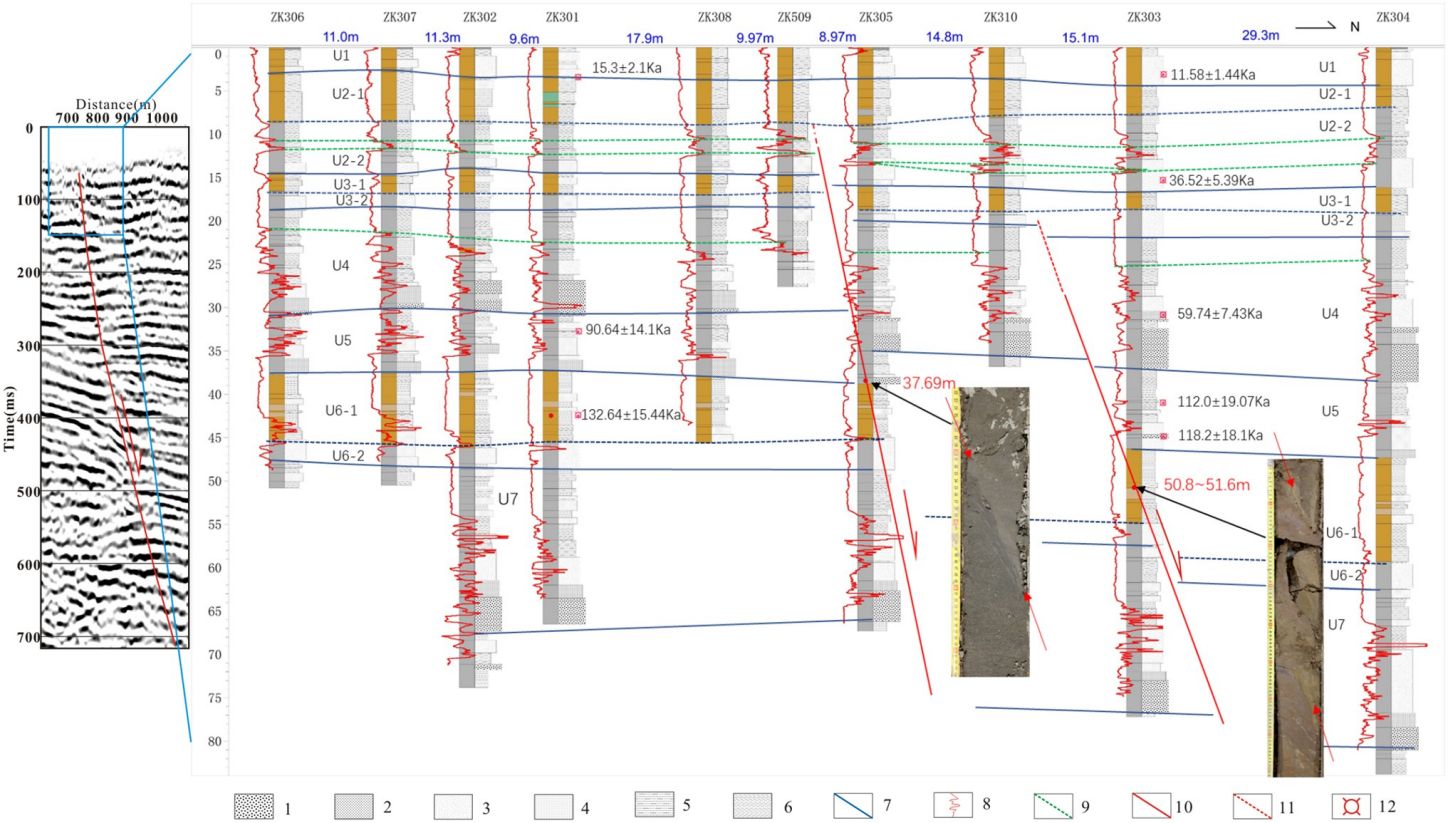
Combined profile of borehole histogram superimposed with magnetic susceptibility logging curve at Xiwugezhuang on L3 Survey line. 1.coarse sand; 2.median sand; 3.fine sand; 4.silt; 5 clay silt; 6.clay; 7 stratigraphic unit boundary line; 8. Magnetic susceptibility curve; 9. Magnetic susceptibility characteristic point line; 10 fault; 11.inferred fault; 12. OSL sample.

The Quaternary strata division scheme, characteristics of the sedimentary cycle, and magnetic susceptibility logging parameters of the nearby standard hole are compared according to the sedimentary lithology. The strata of Xiwugezhuang joint borehole profile can be divided into 7 layers (Fig 6).

U1 layer is mainly composed of light brown silty clay and silty fine sand, having a large number of carbonaceous and rust spots and bands locally. The grain sizes of the sediments increase from top to bottom, and the lithology shows the binary characteristics of "silt-sand", which belongs to a sedimentary cycle. The magnetic susceptibility curve is gentle, and the magnetic susceptibility at the silt and fine sand interface is high. According to the dating results, the Holocene in this layer has been judged.

U2 layer is mainly composed of light brown-light gray silty clay, clay, and silty fine sand, with the majority of fine-grained sediments. The grain sizes of the sediments increase from top to bottom, and the lithology shows the binary characteristics of "silty clay-sand", which belongs to a sedimentary cycle. The light brown silty clay in the upper part is U2–1, and the gray silty fine sand in the lower part is U2–2. The upper part of the susceptibility curve is gentle, and the lower part has several peaks, which is the reaction of the sand layer. According to the dating results, this layer belongs to the late Pleistocene. The lateral thickness of the layer changes slightly, up to about 12 m. The buried depth of the bottom boundary is 13.8–16.51 m. The stratum on the north side is slightly thicker than that on the south side; the thickness of the bottom sand layer also increases.

The U3 layer is mainly composed of brown light gray silty clay, silty clay, and fine silty sand. The grain sizes of the sediments increase from top to bottom. The lithology shows the binary characteristics of "silty clay sand", which belongs to a sedimentary cycle. The brown sandy clay and silty clay at the upper is U3–1, and the light gray silty fine sand at the lower part is U3–2. There is a peak in the sand layer of the magnetic susceptibility curve. This layer belongs to the late Pleistocene strata, with little changes in the transverse thickness that is about 5 m. The buried depth of the bottom boundary is 18.4–21.96 m, and the buried depth of the north side is larger than that of the south side.

The U4 layer is mainly composed of light gray silty clay, clay, and medium-coarse sand. The coarse sand contains gravel, which is sub-angular in shape with the gravel diameter ranging from 0.2 cm to 3 cm. The grain size of the sediments increases from top to bottom, and the lithology shows the binary characteristics of "silty clay-sand", which belongs to a sedimentary cycle. The upper part of the magnetic susceptibility curve is gentle, and the lower part exhibits a serrated jump distribution which is due to the reaction of the sand layer. According to the dating results, this layer belongs to the middle-late Pleistocene strata. Zk305 is used to divide the north and south sides. The stratum on the south side is about 11.5 m thick, and the bottom buried depth is 30.2–30.93 m; the stratum on the north side is about 15.5 m thick, and the bottom buried depth is 35–38.55 m. The stratum on the north side is thicker than that on the south side.

U5 layer is mainly composed of light gray silty clay, silty clay, and silty fine sand, along with a thin layer of medium sand at the bottom, a small amount of rust, carbonaceous spots, and bands in some parts, and a small number of calcareous nodules. There are many peaks in the susceptibility curve, which is zigzag. According to the dating results, this layer belongs to the early-late Pleistocene strata. Zk305 is used to divide the north and south sides. The stratum on the south side is about 7 m thick, and the bottom buried depth is 37.3–38.8 m; the stratum on the north side is about 9.5 m thick, and the bottom buried depth is 46.3–47.32 m, The stratum on the north side is slightly thicker than that on the south side.

The U6 layer is mainly composed of light brown-light gray clay, silty clay, and silty fine sand; in addition, there are thin brown clay and many calcareous nodules. The grain sizes of the sediments increase from top to bottom. The lithology shows the binary characteristics of "clay-sand", which belongs to a sedimentary cycle. The upper part of the light brown silt clay is U6–1, and the lower part of the light gray silty fine sand is U6–2. The magnetic susceptibility curve exhibits gentle characteristics in the upper part and zigzag in the lower part. According to the dating results, this layer belongs to the middle Pleistocene. From zk305 to zk306, the thickness of this layer is about 10.5 m, and the buried depth of the bottom boundary is 47.54–48.71 m. From zk305 to zk303, the thickness is about 12.5 m, and the buried depth of the bottom boundary is 58.82 m. From zk303 to zk304, the thickness is about 16.5 m, and the buried depth of the bottom boundary is 63.5 m. The stratum in the north is gradually thicker than that in the south.

The U7 layer is mainly composed of light gray silty clay, silty clay, fine sand, medium sand, and coarse sand, with a small number of calcareous nodules in some parts. Fine sand has horizontal bedding; the coarse sand contains gravel, which is sub-angular in shape, with the gravel diameter ranging from 0.2 to 1cm. The grain sizes of the sediments increase from top to bottom. The lithology shows the binary characteristics of "silt-sand", which belongs to a sedimentary cycle. The upper part of the susceptibility curve is gentle, and the lower part is zigzag. This layer belongs to the middle Pleistocene. From zk305 to zk306, the thickness of this layer is about 19 m, and the buried depth of its bottom boundary is 66.2–67.33 m. The thickness of the layer from zk305 to zk303 is also about 19 m, but the buried depth of the bottom boundary is 76.88 m. From zk303 to zk304, the thickness is about 18 m, and the buried depth of its bottom boundary is 81 m. The north side is thicker than the south side, and massive changes are observed in the burial depth.

#### 4.2.2 Fault analysis

A fault is found in the core at 37.69 m in zk305, with a dip angle of about 67°. Another fault with a dip angle of 72 ° is found in the core at 50.8–51.6 m in zk303 (photo in Fig 6).

The position of the fault and the buried depth of the upper breakpoint can be seen from the profile of the tandem boreholes. Among them:

The bottom boundary of U7 has obvious dislocation between zk305 and zk303 and between zk303 and zk304; the corresponding displacements are about 9.6 m and 4.1 m, respectively. The bottom boundary of the U6 layer has obvious dislocation between zk305 and zk303 and between zk303 and zk304; the corresponding displacements are about 10.1 m and 4.7 m, respectively. At the bottom boundary of the U5 layer, an obvious dislocation is observed between zk305 and zk303, with a displacement of about 7.5–10.2 m. This is the total displacement of F1 and F2. There is an obvious dislocation between zk309 and zk305 at the bottom of the U4 layer, and the displacement is about 4.5–5.2 m. There is about 1 m dislocation between zk310 and zk303. An obvious dislocation is observed at the bottom of the U3 layer between zk309 and zk305, while the displacement is about 1.8–2.3 m. There is about 1.3–1.7 m dislocation between zk310 and zk303. There is no dislocation at the bottom of U3–1, where the F2 fault extends upward. The U2–1, the upper part of U2, is also continuous, but there are some dislocations at the bottom between zk309 and zk305; the amounts of dislocations are about 1.3–2.9 m. From the connection of the susceptibility logging curve, it can be seen that the connection of the high susceptibility peak in U2–2 is broken, and the amount of dislocation is about 2 m. U1 stratum is continuous, and the phenomenon of fault dislocation is not distinguished.

In conclusion, two faults are found at the Xiwugezhuang row drilling exploration profile, which strikes NE and inclines in NW; their latest active age was the late Pleistocene. It is speculated that the upper breakpoint position of southern fault F1 resides between zk309 and zk305 (116°50′33.82240″, 39°54′ 36.48891″), and the middle part of late Pleistocene strata is staggered. The F2 is located between zk310 and zk303 (116°50′33.69447″, 39°54′37.25392″), with a buried depth of about 18 m. The bottom of the later period of the late Pleistocene strata is faulted. In general, the joint borehole profile exploration at Xiwugezhuang reveals that the latest active age of the Xiadian west fault should be in the later period of the late Pleistocene. According to the U2 dating results of 36.52 ±5.39 ka and its vertical dislocation of 2.13 ± 0.79 m, the slip rate is about 0.058 ± 0.030 mm/a. The dating results of the U5 bottom are 118.20 ±18.10 ka, and the vertical dislocation of the layer due to the fault is about 8.87 ± 1.36 m since late Pleistocene (the difference between the bottom boundary of U5 layer of zk306 and zk304 holes). The vertical slipping rate of the fault has been about 0.075 ± 0.023 mm/a since the late Pleistocene. Due to the thick Quaternary overburden in the area, fault dislocation is decaying upward, and the upper slip rate may be less than that of the deep fault. The results show that the slip rate since the late Pleistocene is lower than that since the late Pleistocene. One possible reason could be that the activity intensity of the fault since the late Pleistocene is lower than that since the late Pleistocene. Another reason may be that the dislocation depth of the late Pleistocene strata is greater than that of the late Pleistocene strata, and the dislocation rate in the shallow part is less than that in the deep part.

## 5. Discussion

### 5.1 Nature of the Xiadian west fault and its relationship with the Xiadian fault and Daxing Fault

Our results shows that the Xiadian west fault, which is probably a branch of the Daxing Fault, is a northwest-dipping active fault between the Daxing fault and Xiadian fault. The Xiadian fault, Xiadian west fault and Daxing fault form a large fault zone with NE strike and right-step echelon. The Daxing fault is directly connected to the Xiadian fault from the profile in Fig 2. This result coincisdes with the opinion of Gui et al. [31] and Zhang et al. [35], which suggests that the north section of the Daxing fault tends to be directly connected with the Xiadian fault. The Xiadian west fault controls a half-graben-type sag, which has not been described before. It is a newly discovered small sag in the northwest direction of the Dachang depression, mainly located in Yanjiao Economic and Technological Development Zone of Sanhe City, which is called Yanjiao Sag in this paper. The sag is deep in the South and shallowed in the north. The thickest part of Neogene and Quaternary can reach up to 600 m, while the thickest part of Quaternary can reach 300 m(Fig 3). The maximum vertical dislocation of the Neogene by the Xiadian west fault is 65 m. The vertical dislocation since the middle Pleistocene is approximately 20 m.

The Xiadian west fault inclines to the northwest, opposite the Xiadian fault. The distance between them varies massively. The distance between them is wide in the south and close in the north. A horst is sandwiched between the two faults. By analyzing the results of deep seismic exploration (Fig 2) [11,28,44], it can be concluded that the Xiadian fault, Xiadian west fault and Daxing fault belong to the same deep fault, which is the branch of the deep activity of the deep fault in the shallowed part of the crust. They may have formed an echelon assemblage on the plane, forming a large fault zone. Together, they may form a large earthquake structure or act as independent seismogenic structures. Xiadian fault is the seismogenic structure of the 1679 Sanhe-Pinggu M8 earthquake. The fracture of the Xiadian fault, which has 30 km length and 10 km surface fracture zone [1,2,14,18], is not consistent with the fracture size of a magnitude 8 earthquake [27]. As a branch of Daxing Fault, the motion characteristics of the Xiadian west fault are very similar to those of the Xiadian fault. Xiadian west fault is close to the Xiadian fault in space and is closely related to the Xiadian fault in the deep structure. These faults are echelon assemblage and constitute a large fault zone. The Xiadian west fault, as part of this large fault zone, is probably part of the seismogenic structure of the Sanhe-Pinggu M8 earthquake in 1679.

### 5.2 Problems and suggestions

This study is only limited to the Sanhe area. The fault enters southward to Tongzhou, Daxing and Langfang. In those areas, further investigations are needed to identify the activity of the fault. The role of the fault and the Xiadian fault in the seismogenic environment also needs further study. The fault is located in the sub-center of Beijing. Moderate strong earthquakes occurred along the line. This work also provides evidence of its activity since the late Pleistocene. Special attention should be paid to the potential seismic risk of the fault. Due to insufficient information and data, this paper does not evaluate seismic risk analysis, but it is urgent to carry out further work on the fault.

## 6. Conclusion

The plane distribution of the three reaches of the Xiadian west fault can be preliminarily determined by the fault breakpoints that are obtained in six shallow seismic profiles. The fault is sinuous, and the strike is NE in general, which shows the nature of the normal fault. According to the joint borehole profile and dating results, the latest active age of the Xiadian west fault is determined as the later period of the late Pleistocene. Since the later period of the late Pleistocene, the vertical slip rate has been about 0.058 ± 0.030 mm/a. Since the late Pleistocene, the vertical slip rate has been about 0.075 ± 0.023 mm/a.
